# Integrated environmental DNA analysis and population assessment revealed a biannual breeding season of the Korean clawed salamander (*Onychodactylus koreanus*)

**DOI:** 10.1371/journal.pone.0342469

**Published:** 2026-02-05

**Authors:** Min-Woo Park, Jaejin Park, Jongsun Kim, Jiho Park, Narae Joo, Hahyun Nam, Daesik Park

**Affiliations:** 1 Interdisciplinary Program in Earth Environmental System Science & Engineering, Kangwon National University, Chuncheon, Gangwon, Republic of Korea; 2 Division of Science Education, Kangwon National University, Chuncheon, Gangwon, Republic of Korea; Charles University: Univerzita Karlova, CZECHIA

## Abstract

Determining the breeding season of a species is key to understanding its life cycle and to ensuring efficient conservation. Determining the breeding season is challenging for subterranean breeders. Environmental DNA (eDNA) applications have been proposed alongside population surveys. In this study, larval influx, adult emergence, and eDNA detection of *Onychodactylus koreanus*, an external fertilizer, were investigated at underground cave breeding sites every 2 weeks from April 2024 to June 2025 to determine the breeding frequency and season. A major influx of 1-year-old larvae occurred in June and November. Adult emergence occurred in May–June and November–December, but was absent in July–September and February. The timing of major eDNA detection matched the pattern of adult emergence, but did not well reflect the larval influx. Major eDNA detection occurred twice, in April–June and November–December 2024. Three egg clutches were observed on December 23, 2024. On July 28, 2025, 110 eggs were counted at the same spawning site. Our combined results suggest that *O. koreanus* has two sperate breeding seasons each year, occurring April–June and November–December, and that eDNA detection along with population surveys can be useful to identify the breeding season of subterranean amphibians.

## Introduction

The amphibian breeding season is influenced by various proximate factors, including temperature, photoperiod, and nutritional status, as well as genetic traits [1–2]. The breeding periods of amphibians are largely continuous or periodic [3–4]. However, they may have multiple breeding periods within a year [3,5]. Identification of an accurate breeding period is the starting point for understanding the overall life history of a species and provides key information for its efficient conservation. If multiple breeding events occur within a year, they can induce reproductive isolation among individuals and may serve as a starting point for sympatric speciation [6–8]. Despite the importance of this knowledge, the breeding seasons of some species in specific environments remain unknown, making their determination challenging.

The breeding period of amphibian species can be identified through direct surveys of breeding adult emergence at the breeding site and observation of mating and egg laying. Most amphibians spend their non-breeding periods in terrestrial habitats and enter water bodies to mate and lay eggs during the breeding season [5]. Determining the breeding period is challenging when breeding occurs underground. Breeding periods have been determined in fish such as bass, carp, and lamprey using environmental DNA (eDNA) analysis [9–11]. In these studies, eDNA detection showed a positive correlation with the number of fish at the site; in particular, eDNA concentration increased during spawning and fertilization. In externally fertilizing amphibians, breeding females enter the water only for mating activity, whereas males remain in the water for some time [12]. Recent studies on seasonal eDNA fluctuations in *Triturus cristatus* [13] have shown an increase in eDNA during the breeding season of *Cryptobranchus alleganiensis* [14], and seasonal changes in eDNA detection in *Hynobius kimurae* [15] have revealed the applicability of eDNA analysis in the study of amphibian reproductive ecology. In particular, to overcome subterranean conditions where direct individual observation is limited, eDNA analysis methods exhibited potential [16–17]. Real-time TaqMan polymerase chain reaction (PCR) using mitochondrial DNA (mtDNA) primers is commonly used in eDNA monitoring studies. However, the mtDNA: nuclear DNA ratio has been considered only recently in fish [11]. Recent studies have revealed that combining eDNA analysis and population surveys could further enhance the reliability of the [13,18].

The exact breeding frequency and period of species in the genus *Onychodactylus*, which inhabits the mountain valleys of Northeast Asia and breeds underground through external fertilization [19], remain largely unknown. To date, 12 species have been identified. One species is from the Russian Far East, two from the People’s Republic of China or the Democratic People’s Republic of Korea, two from the Republic of Korea, and seven from Japan [20–24]. *Onychodactylus spp*. have degenerate lungs and primarily rely on their skin for respiration; therefore, they only inhabit moderate-altitude, densely forested, cooler mountain valleys with high dissolved oxygen concentrations [25]. Adults spend non-breeding periods in the streamside forests of mountain valleys and enter streams during the breeding period [25]. Female *Onychodactylus* lay eggs by attaching them to rocks or tree roots in underground spaces where water springs are located, and males grab egg sacs to externally fertilize the eggs by spreading their sperm [19,26,27]. These two egg sacs contain 9–22 eggs [19,27]. The eggs hatch over approximately 3–5 months [28–29], and the hatched larvae spend approximately 2 years in the streams, metamorphose during September, and move to the streamside forest [30]. Because of the secretive life history and subterranean breeding of *Onychodactylus*, its breeding frequency and period are not precisely known. The lack of such information is a limiting factor in the study of life history, habitat adaptation, and species differentiation within this genus. Moreover, the recent intensification of droughts owing to climate change has altered the hydrological environment in high-altitude areas, further threatening *Onychodactylus* conservation [31–35]. For example, *O. fischeri* is an endangered species in Russia [36]. *Onychodactylus zhangyapingi* and *O. sillanus* have minimal distribution ranges, making their conservation challenging [34,37]. Therefore, to understand their life histories and conserve them efficiently, it is necessary to accurately determine their exact breeding frequencies and periods.

However, the annual breeding frequency of *Onychodactylus* remains poorly understood. Breeding is likely to occur more than twice annually, although breeding once a year cannot be ruled out. The mating and spawning of *Onychodactylus* have only been directly observed once in an underground cave [19]. Observations of eggs laid are sporadic across countries [27,38]. In *O. fischeri*, breeding adults appear from May–June in the breeding stream, but a small number also appear in September [39], indicating the possibility of breeding twice. In contrast, Serbinova and Solkin [40] considered the likelihood of breeding during cold winters to be low and proposed annual breeding for *O. fischeri*. In the Sikhote-Lain area, eggs are laid in late May [27]. In the case of *O. japonicus*, breeding adults largely move to breeding sites in May–June and October–November on Hodatsu Mountain [41]. Hayase and Yamane [29] showed that on Tsukuba Mountain, a major influx of newly hatched larvae appeared more than twice annually. These results reveal that *O. japonicus* breeds more than twice annually. In *O. koreanus*, the influx of 1-year-old larvae occurred in mid–March and October–November, but eggs were laid only in June [19,30]. To clearly reveal the two breeding seasons of *Onychodactylus spp.*, it is essential to confirm the emergence of breeding adults, particularly ovulated females, and the presence of eggs laid during two separate periods annually within a breeding site. Hwanseongul Cave, where direct mating and *O. koreanus* eggs can be observed [19], provides a unique study site to resolve this question.

The Hwanseongul Cave, where massive mating and spawning of *O. koreanus* were observed in 2005, is open and accessible for research. In this study, to determine the frequency and period of the breeding season of *O. koreanus*, we surveyed larval influx, adult emergence, and eDNA in caves twice monthly between April 2024 and June 2025.

## Materials and methods

### Field survey

This study was conducted between April 21, 2024, and June 20, 2025, inside Hwanseongul Cave (37°19’28.13“N; 129°29’29.81”E) in Samcheok City, where the *O. koreanus* mating site is located [19]. The cave is the type locality of *O. koreanus*, and in the cave [22], other *Onychodactylus* species are absent based on survey and genetic studies [42–43]. On July 29, 2025, we visited the cave to confirm egg laying in 2025. Hwanseongul Cave is the largest limestone cave in the Republic of Korea, estimated to be approximately 8,500 m in length. The inside temperature is relatively constant at 6.5–13.5 °C, unlike the outside temperature, which ranges from −23.3–32.4 °C throughout the year [44]. The cave receives rainwater from a rural village located 500 m above it [44]. The study site is the only location in Northeast Asia where the mating and spawning of *Onychodactylus* species can be directly observed [19]. As water does not freeze in winter, it enables year-round population surveys, making it highly suitable for breeding ecology studies of subterranean breeders. During the study period, surveys of larval influx and adult emergence in the two areas and eDNA sampling at the two sites were conducted every 2 weeks. Survey and eDNA samples were collected 29 times.

**Fig 1:**
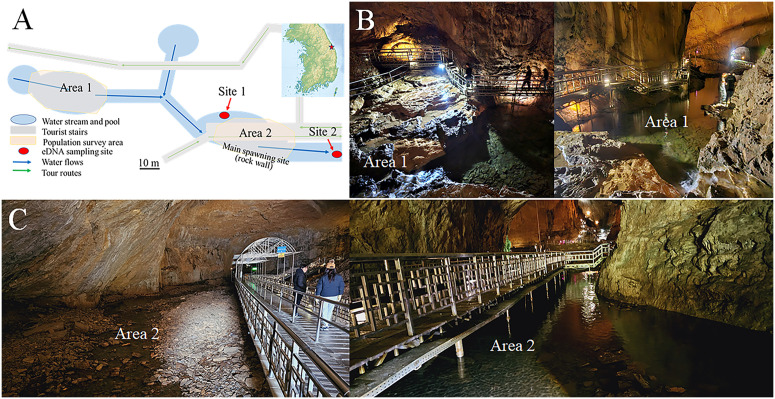
Population survey areas (Areas 1 and 2; B, C) within the Hwanseongul Cave, where mating and egg laying of *Onychodactylus koreanus*, a subterranean breeder, were previously observed. The environmental DNA (eDNA) sampling sites are also shown in the schematic diagram (A).

A population survey was conducted, with the first area surveyed from May 30, 2024, and the second area surveyed from April 21, 2024. The first area included a water pool approximately 20 m long and up to 2 m deep with small watercourses flowing into the pool (Fig 1). Two colleagues walked slowly around the edge of the pool and watercourses and counted the larvae and adults according to the methods outlined below. The second survey area was located approximately 50 m downstream of the first (Fig 1). It includes the known primary spawning site near the pool and the stream below the spawning site. The total length was approximately 30 m. This area is located approximately 60 m from the cave entrance. In the second area, we attempted to identify individuals by turning stones over inside and around the stream, as well as by counting exposed individuals. We selected these two areas because we could observe individuals without disturbing their habitats. The first area was upstream of the first and second eDNA sampling sites, and the second was located between the two eDNA sampling sites (Fig 1).

An individual survey was conducted by two colleagues for approximately 20 min after eDNA sampling. Larvae and adults were counted separately. Because of the water depth in the surveyed areas, it was not possible to capture adults or larvae in most areas. In addition, the number of larvae often exceeds 50, making it impractical to capture all individuals in every survey. Therefore, we classified the larvae into the < 1-year-old group and the > 2-year-old group based on a total length of approximately 5 cm [29–30]. Our size grouping into two classes may be useful for determining the influx time of newly hatched larvae. In addition, we recorded the time at which larvae with yolks appeared and tracked their absorption of the yolks because *Onychodactylus* larvae can appear with or without egg yolk [21]. Two researchers practiced class prediction together to achieve mutual agreement on size classification and conducted all surveys. For adults, we classified them into sub-adults with a total length of 10 cm or less and adults with a total length of >10 cm [29]. Given our multiple surveys, we attempted to minimize disturbance to the habitat. We determined the sex of adults based on their cloacal shape [21]. Females were further classified as ovulated or oviposited females based on the presence of visible eggs in their abdomens. Considering that we surveyed a breeding population, classifying females into two groups was reasonable [12]. When we could not capture but observed the adults, we determined their sex as male when a broad tail, thick toe, and extended lateral folds on the hind foot edge were visible [45,46]. Females with visibly ovulated eggs were classified as ovulated females, and in other cases, as oviposited females.

All animal sampling and handling procedures were conducted in accordance with the guidelines of the Society for the Study of Amphibians and Reptiles (SSAR) and the American Society of Ichthyologists and Herpetologists (ASIH) for the use of live amphibians in the field [47]. The study and sampling in Hwanseongul Cave were made possible with the permission and support of Samcheok City Hall and the Korea Heritage Service (Samcheok 202501). The sample collection and all survey procedures were approved by the Institutional Animal Care and Use Committee (IACUC) of Kangwon National University (IACUC approval number KW-240603–1).

### eDNA sampling and extraction

**Fig 2:**
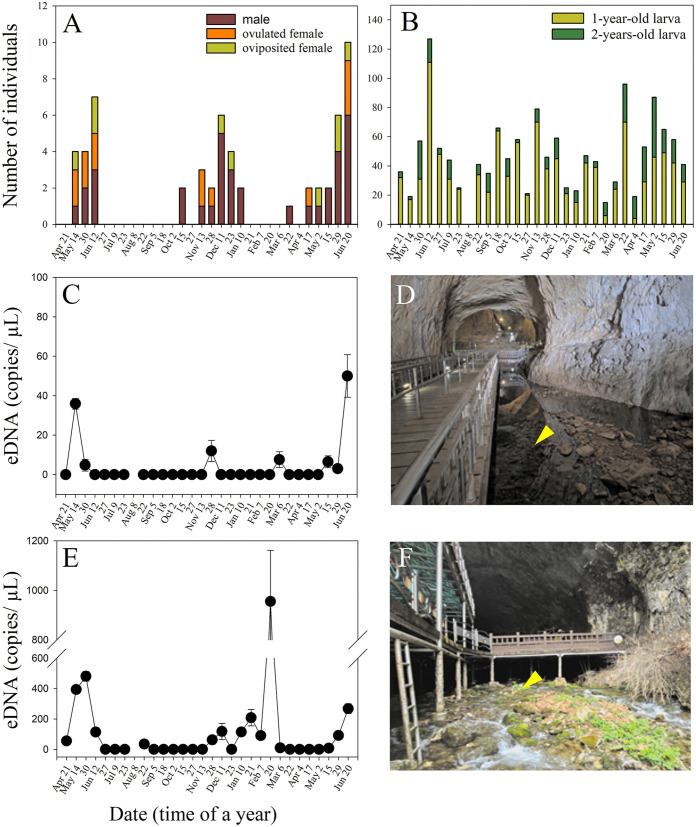
Population survey of adults (A) and larvae (B), and environmental DNA (eDNA) detection of *Onychodactylus koreanus* at the first (C) and second (E) sampling sites over 14 months between April 2024 and June 2025. The first site (D) was approximately 60 m inside the cave, and the second site (F) was at the cave entrance.

eDNA sampling was conducted at two sites (Figs 1 and 2): the first site was near the point where spawning was previously observed in 2005, and the second site was around the cave entrance where water flowed out. The first site was located in a pool, slightly away from the primary flow of the stream and opposite to the known primary spawning site, at a depth of approximately 0.5–1 m. The second site is located where water from inside the cave gathers into one of the four or five watercourses and flows out. The eDNA sampling was conducted before each population survey. Water was collected in a clean plastic container (3 L), and 1 L of water was filtered through a Sterivex filter (pore size, 0.45 μm, Millipore, Burlington, MA, USA) using a 50 mL syringe. Sampling was performed in duplicate. Thereafter, the water was removed, and the filter was filled with 99.5% EtOH, then sealed with Parafilm. New gloves, water bottles, syringes, and sampling kits were used at each site to prevent DNA contamination. On each sampling day, 1 L of commercial bottled water was filtered on-site using the same method to serve as the control sample. The eDNA samples were stored at −20 °C until further analysis.

eDNA was extracted from the Sterivex filters using the DNeasy Blood & Tissue Kit (Qiagen, Hilden, Germany), as described by Kawato et al. [48]. In brief, the Sterivex filter cartridge was removed using a pipe cutter (Smart Spring Tube Cutter, Black Diamond, Taichung, Taiwan), and the 99.5% EtOH was removed using the RNAlater removal method. The detached filter was shredded to small fragments; lysed in 400 µL phosphate-buffered saline (PBS; pH 7.0), 400 µL Buffer AL, and 40 µL proteinase K per filter; incubated at 56 °C for 2 h; and finally eluted using 200 µL elution buffer per filter [48]. The extracted eDNA was measured using a NanoDrop (NanoDrop Lite Plus, ThermoFisher Scientific, Waltham, MA, USA) and stored at −80 °C (VS-90DF-C Series, VISION SCIENTIFIC, Bucheon, Republic of Korea) until further use.

### Developing a species-specific TaqMan probe and primer set

To develop species-specific primers and probes, three partial mitochondrial COI sequences (GenBank accession No. JX158160, JX158161, and JX158162) of *O. koreanus* were obtained from the National Center for Biotechnology Information (NCBI) GenBank (https://www.ncbi.nlm.nih.gov/genbank/) and used as reference sequences. The primer developed for *O. japonicus* [49] detected both *O. koreanus* and *Hynobius leechii*, which cohabit Korean mountain valleys. Therefore, we developed new primers using the Primer 3 plugin Geneious Prime v.21.0.4. (https://www.geneious.com). To increase the sensitivity of quantitative PCR (qPCR), we selected primer and probe sequences that did not form self-dimers or hairpin structures. We set the annealing temperature of the probe as 5°C higher than that of the primer. This was the highest temperature that could be set using the reference sequences as suggested [50].

We performed in-silico tests of the primer and probe sets using 168 COI sequences from 11 amphibian and seven reptile species (S1 Table) that have the possibility to cohabit with *O. koreanus* in Korean mountain valleys. Next, an in-silico test was also conducted using the *PrimerMiner* package in R [51], and considered that if the penalty score of either the forward primer, reverse primer, or probe exceeded 120, amplification of the specific sequence failed [52]. Based on the results, we selected the forward primer OK_eDNA_CO1_F (5’–AGCCTCATCAGGAGTTGAAGC–3’), reverse primer OK_eDNA_CO1_R (5’–TCTACAGAAGCTCCGGCATG–3’), and probe OK_eDNA_CO1_probe (5’–FAM-AGGATGAACTGTTTATCCTCCTCTAGCAGG-BHQ1–3’), which specifically detected the COI of *O. koreanus* (93 bp).

To determine the optimal annealing temperature of the primer and probe set, we tested three different temperatures (57 °C, 60 °C, and 63 °C) using PCR (SimpliAmp Thermal Cycle, Thermo Fisher Scientific, Waltham, MA, USA) based on the Tm value of 60 °C from Geneious Prime following the previous study [53]. We used the genomic DNA of *O. koreanus* (G05249OK, G05250OK) collected from the type locality (Hwanseongul Cave) of the species. As a result, we selected 60 °C, which was initially suggested by Geneious Prime, because the three conditions yielded similar amplification results, as confirmed using 1% agarose gel electrophoresis (S1 Fig). In addition, to determine the appropriate amounts of the probe in qPCR runs, we tested three different ratios between probe and primer (150, 200, and 250 nM probe vs. 500 nM primer), selected the best ratio based on the lowest CT value, and applied the ratio in subsequent qPCR analyses (S2 Table). Subsequently, the amplification ability of the primers and probes was tested in vitro using tissue DNA from nine amphibian and three reptile species (S3 Table, S2 Fig) using qPCR. Among them, two species, *Hynobius leechii* and *Rana uenoi*, were previously reported in the Hwanseongul Cave [42].

To determine the limit of detection (LOD) and limit of quantification (LOQ) of the primer and probe, we conducted five replicates of qPCR using a 10-fold serial dilution of gBlock, ranging from 10^9^–0.1 copy/µL (176 bp, Integrated DNA Technologies, Coralville, IA, USA; S4 Table). The LOD and LOQ were calculated using the *drc* package in R following a previous study (S5 Table, S3 and S4 Fig) [54]. Finally, we confirmed that the primer and probe correctly amplified the targeted *COI* sequence of *O. koreanus* in field eDNA samples. For this, we collected 1 L of water from three different mountain valleys (Goeun, Doekduwon, and Neuratjae) in Chuncheon, Kangwon, where larvae of *O. koreanus* were present. eDNA was extracted from the samples, and qPCRs were run as described below (S6 Table). The qPCR products were sequenced by Macrogen (Seoul, Republic of Korea). We visually inspected and aligned the obtained sequences using MUSCLE [55] and trimmed the sequences using Geneious Prime v2022.0.2 (https://www.geneious.com). For species identification, we used the Basic Local Alignment Search Tool (BLAST) within NCBI in Geneious Prime.

### eDNA analysis using qPCR

To detect and quantify the eDNA of *O. koreanus* in the eDNA samples, qPCR using a TaqMan probe was performed using a QuantStudio 1 Real-Time PCR (Thermo Fisher Scientific, Waltham, MA, USA). The amplification solution used for qPCR was as follows: 500 nM of forward primer (1 μL), 500 nM of reverse primer (1 μL), 200 nM of TaqMan probe (0.4 μL), 10 μL of TaqMan Environmental Master Mix 2.0 (Thermo Fisher Scientific, Waltham, MA, USA), 4 μg of molecular biology-grade bovine serum albumin (BSA; Thermo Fisher Scientific, Waltham, MA, USA), and 4 μL of eDNA template. The total volume was adjusted to 20 μL with molecular biology grade water (HyClone, Waltham, MA, USA). Temperature conditions for qPCR were as follows: 10 min at 95 °C, 40 cycles of 15 s at 95 °C, 30 s at 64 °C, and 30 s at 60 °C. We set the extension temperature at 60 °C following the manufacturer’s instructions (Thermo Fisher Scientific, Waltham, MA, USA). We included the BSA in the amplification solution because it successfully suppresses inhibitors in qPCRs using a TaqMan probe master mixture [56]. In our preliminary experiments, the addition of 4 µg of BSA yielded better performance in our qPCR runs; therefore, we used the same amount. All experiments included gBlock standards (10^4^, 10^3^, 10^2^, and 10 copies/ μL) for quantification, a field negative control, sampled and extracted using the same process, and a no-template control. All the PCRs were performed in triplicate per sample. When the detection was observed in only one of the three replicates, or the detection was not observed in any replicates, we performed qPCR of the samples again. The qPCR results were analyzed using Design & Analysis Software version 2.7.0 (Thermo Fisher Scientific, Waltham, MA, USA). If eDNA was detected but not quantifiable (<LOQ), we sequenced the qPCR product and used its concentration only if *O. koreanus* was identified in it. In the case of no eDNA detection, the concentration was considered 0. For the DNA concentration in a sample on a specific day, we calculated the average DNA concentration of six replicates (duplicate samples × triplicate qPCR runs). During the qPCR analyses of the experimental samples, we confirmed whether we correctly detected the eDNA of *O. koreanus* in 22 experimental eDNA samples (S6 Table) through sequencing, as described above.

The eDNA detection frequencies between the two eDNA sampling sites were compared using the chi-square test. The detection amounts were compared between the two sampling areas during the sampling period using a repeated measures general linear model (GLM) analysis. In addition, the relationships between the number of larvae and adults and the amounts of daily eDNA detection were analyzed using Spearman’s correlation test because most data did not pass the Kolmogorov-Smirnov test (*P* < 0.05). All statistical analyses were done in SPSS (ver. 29.0.2.0).

### Species determination of adults and larvae

Genetic tissue samples were collected on January 10, 2025, to determine the species of the surveyed individuals that hatched at different times of the year. The tail tips of six adults and 14 larvae were cut and stored in 95% ethanol. To cover all ages of the individuals surveyed, we sampled newly hatched small-sized larvae (winter–born group), mid-sized larvae approximately 6 mm TL (summer–born group), large-sized larvae more than 2-years-old, and adults. To confirm whether they are genetically the same species, we investigated mitochondrial COI, which is often used for DNA barcoding of salamanders [57]. DNA was extracted from the tissue using the DNeasy Blood & Tissue Kit (Qiagen, Hilden, Germany) and stored at −80 °C until further analysis. Partial sequences of the COI gene were amplified using the LC01490 and HC02198 primers [58]. PCR was performed using a SimpliAmp Thermal Cycler (Applied Biosystems, California, USA) in a volume of 20 μL, which consisted of 5 ng of template DNA (1 μL), 500 nM each of forward and reverse primers (each 0.5 μL), and 10 μL of 2X TOPsimple™PreMIX-nTaq (Enzynomics, Daejeon, Republic of Korea), and finally adjusted with molecular biology grade water (HyClone, Waltham, MA, USA). The PCR products were confirmed using a 1% agarose gel and sequenced by Macrogen (Seoul, Republic of Korea). We visually inspected and aligned the obtained sequences using MUSCLE [55] and trimmed them using Geneious Prime v.2022.0.2 (Biomatters, Auckland, New Zealand). For species identification, we used the method described above. In addition, we identified nucleotide mutations in COI using DnaSP v.6 [59] and determined its haplotype using a combined reference and obtained COI sequences of *O. koreanus*.

## Results

### Population survey

Most adults and larvae of *O. koreanus* were present on the floor and rock face inside the stream and pool in Hwanseongul Cave. No other amphibian species were observed in the cave. As the population survey results were similar between the two survey areas, we combined the data. Larval influx and adult emergence based on each survey area are presented in S7 Table and S5 Fig. Over the two areas, we recorded 48.7 ± 25.4 SD *O*. *koreanus* larvae (range: 15–127 larvae) in each survey (Fig 2B): 1,092 1-year-old (37.7 ± 21.9 SD) and 318 2-years-old larvae (11.0 ± 9.2 SD). In 2024, the major influx of 1-year-old larvae occurred on June 12 and November 13, with 127 and 79 larvae, respectively, and in 2025, with 96 larvae on March 22 (Fig 2B). The period of larval influx was longer in autumn (September–February) than in late spring (April–June). Larvae with yolks were observed on November 13, 2024, and until January 10, 2025. In both years, no larvae with egg yolks were observed between April and October.

**Fig 3:**
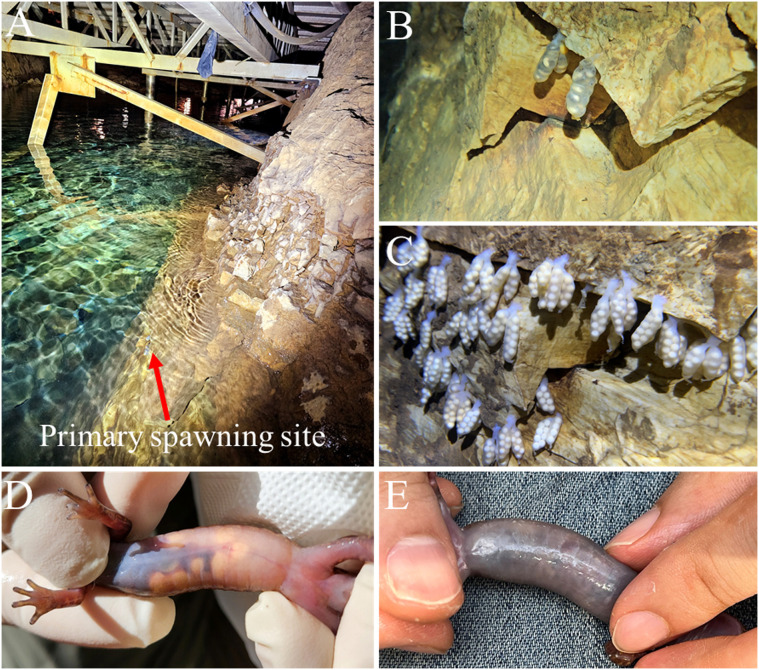
Primary spawning site (A) of *Onychodactylus koreanus*, a subterranean breeder, and their eggs observed on December 13, 2024 (B) and those recorded on July 28, 2025 (C). The primary spawning site is located on the rock face underwater, indicated by a red arrow (A). At the bottom, ovulated (D) and oviposited (E) abdomens of females are shown.

The major emergence of breeding adults occurred between May and June and between November 2024 and January 2025 (Fig 2A). The adults were recorded on March 22, 2025. The adults (n = 2) confirmed on October 15, 2024, were recently metamorphosed small adults and were treated as males because sex determination was not possible. Overall, we counted 36 males and 22 females. Ovulated females were observed during the three adult emergence periods (Fig 2A). Of the 22 females, 13 ovulated (Fig 3D) and nine oviposited (Fig 3E). The abdomens of oviposited females were relatively swollen, but no eggs were visible.

On December 23, 2024, three egg clutches were observed at the primary spawning sites (Fig 3A and 3B). One was not fertilized. The two egg clutches contained 15 (eight and seven eggs per egg sac) and 14 (eight and six eggs) eggs, respectively. The eggs hatched on May 2, 2025. On July 28, 2025, 110 egg clutches were observed at the same site (Fig 3C). Examined clutches contained 16.6 ± 1.1 SD eggs (range: 15–18 eggs, range: 5–9 eggs per egg sac, n = 10). We recorded 16 dead larvae on the watercourses and dried rocks in the first survey area on January 21, 2025. In the follow-up survey 2 weeks later, no additional deceased individuals were recorded, but they were decomposing on-site.

### eDNA detection

In-silico tests, developing a primer set did not amplify any of the 168 sequences (S1 Table) or the non-target genes in the primer specificity test in Geneious Prime. In vitro tests, only the genomic DNA of *O. koreanus* and *O. sillanus* showed positive results. The cycle threshold (CT) values of them significantly differed (S2 Fig). *Onychodactylus sillanus* does not inhabit our study area [42], as its distribution is limited to the southeastern parts of the Korean Peninsula [23]. The Hawansungul cave is also the type locality of *O. koreanus*, as stated above [22]. Therefore, we moved forward with our primer and probe development. The 1: 2.5 ratio of probe to primer showed the best CT results (S2 Table); therefore, we included 200 nM of the probe in the amplification solution. The LOD and LOQ of the primer were 4.52 copies/ µL (S4 Fig). In the field of eDNA sample tests, the target DNA from all three samples was successfully amplified and confirmed to be *O. koreanus* through sequencing (S6 Table). We did not detect any eDNA from 80 samples, but detected eDNA above the LOD in 34 samples and below the LOD in 2 samples. In the second qPCR tests, we detected eDNA in three replicates of one below-LOD sample but none in the other sample or in the 80 samples. Among the amplified three replicates, one detection was below the LOD (3 copies/ µL). Through subsequent sequencing, we confirmed it as *O. koreanus*, so we included it in subsequent analyses. Furthermore, we confirmed that we correctly detected the eDNA of *O. koreanus* in the 22 experimental eDNA samples by sequencing (S6 Table). In the analyses, none of the field and NTC controls showed positive responses.

The amounts of eDNA detected varied based on sampling sites (F_28,280_= 23.7, *P* < 0.01), and the interaction between sampling site and time was significant (F_28,280_= 22.2, *P* < 0.01). We detected eDNA above the LOQ on 9 of 15 d when *O. koreanus* females or males appeared at the breeding site (Fig 2C and 2E, S8 Table). We detected eDNA above the LOQ on 5 d when we did not observe breeding adults between January 21 and February 7, 2025, with the exception of 1 d (October 15, 2024), when recently metamorphosed adults were detected (Fig 2C and 2E, S8 Table).

At the first sampling site, we detected the eDNA of *O. koreanus* above the LOQ in 7 of 29 daily samples. At the second outlet site, 15 of the 29 daily samples showed positive results. Major eDNA detection occurred at the first site: on May 14 and November 28, 2024, and on March 6 and June 20, 2025. At the second site, major eDNA detection occurred on May 30, December 11, 2024, and January 21, February 20, and June 20, 2025 (Fig 2C and 2E, S8 Table). Overall eDNA detection rate at the second site (73 of 174 replicates) was higher than that at the first site (26 of 174 replicates; Chi-square test, df = 1, *P* < 0.01).

The amounts of eDNA detected daily (n = 29) at the first site samples (4.1 ± 1.1 SE copies/ µL; S8 Table) show the positive relationship with those at the second site (103.6 ± 18.2 SE copies/ µL; S8 Table) (r = 0.455, n = 29, *P* = 0.013). The amounts of eDNA detected at the first site were significantly correlated with the number of breeding adults (r = 0.442, n = 29, *P* = 0.016) but not with that of larvae (*P* > 0.05). Other comparisons were not significant (*P* > 0.05). The amounts of the extracted DNA and eDNA detected at each sampling site are presented in S8 Table.

### Determining the species

Seven small-size larvae (total length: 3.46 ± 0.24 SD cm, range: 3.3–3.9 cm), two mid-size larvae (4.67 ± 0.39 SD cm, 4.5 and 4.9 cm), five large-size larvae (6.51 ± 0.84 SD cm, 5.5–7.9 cm), and six adults (17.6 ± 1.4 SD cm, 15.4–20.5 cm) were all identified as *O. koreanus*. Sixteen individuals, excluding one small larva, one large larva, and two adults, had the same COI haplotype (GenBank accession No. PX204951–PX204955 and PQ932564).

## Discussion

Owing to secret subterranean breeding, the breeding frequency and period of *Onychodactylus spp*. remain debatable [29,30,39–41]. In this study, we surveyed and analyzed larval influx, adult emergence, and eDNA in the subterranean breeding population of *O. koreanus* over a period of 14 months. Our results suggest that *O. koreanus* breeds twice annually, in April–June and November–December. Our results raise research questions regarding the occurrence of breeding twice annually.

In the present study, the emergence of ovulated and oviposited females at the breeding site occurred twice annually, in May–June and November–December of 2024. In all emergence cases, males and ovulated/oviposited females were observed together. Females in externally fertilizing salamanders appear in the breeding population to lay their eggs [12]; finding both ovulated and oviposited females suggests that spawning occurred. In breeding populations of *O. japonicus* and *O. fischeri*, breeding adults have been observed twice annually, in late spring and autumn or early winter [41,45]. In our study, adults were not observed during July–September or February. This absence pattern has been partially confirmed in *O. japonicus* [41]. Overall, adult emergence patterns at the breeding sites confirmed in this study were similar to those reported in previous studies [41,45], with two emergences annually in late spring and early winter. In our study, the influx of 1-year-old larvae into the primary stream occurred twice annually, in April–July and September–December, 2024. In a previous study on *O. koreanus* larvae, a 1-year-old larval influx was observed twice, in November and March [30]. In *O. japonicus*, larvae of three different sizes are present within the stream in April and August [29]. These results indicated that major influx of newly hatched larvae occurs twice annually. During our survey, newly hatched larvae with yolks were noted from November 13 to January 20; however, we did not observe such larvae from April to October. Larvae can appear with or without egg yolk in the primary stream [21], suggesting that the influx time of 1-year-old larvae can be varied. New larval influx into the primary stream after hatching may depend on various factors, such as microhabitat heterogeneity, which shows different focal temperature conditions and affects the hatching period of larvae, and the water velocity at each spawning site, which facilitates the active or passive movement of larvae in the habitats [29–30]. The overall 1-year-old larval influx pattern of *O. koreanus* was similar to that reported in previous studies; however, the influx size varied depending on the survey area and time of year.

Our eDNA analysis reflected adult emergence in the breeding population. In amphibians, the amount of eDNA obtained from a single survey generally reflects the number of individuals in the survey area [14,60,61]. However, long-term eDNA detection results in significant variation, making it difficult to assess population fluctuations [13,18]. Our proposed eDNA detection method has several implications. First, the timing of major eDNA detection did not align well with larval influx. For example, although larvae were present from July to September 2024, positive eDNA was not detected. Despite the LOQ of our primer being 4.52 copies/ μL, which is similar or slightly higher than 1–15 copies/ reaction in the previous studies [60,62], the eDNA detection of larvae was low, which can be attributed to two reasons. 1) The amount of eDNA released by larvae may be significantly lower than that released by adults. In *Salamandrella keyserlingii*, the amount of eDNA leased from ten larvae was lower than that released from two adults [62]. Considering the streamlined, slender body shape and adaptation to fast water currents of *Onychodactylus* larvae [25], eDNA release may be further reduced. eDNA detection of larvae may effectively provide information about the population’s existence in a mountain valley when population surveys are limited or population density is very low. Therefore, characterizing their eDNA release and developing more sensitive nuclear markers are necessary [63–64]. 2) The overall low eDNA detection rate observed in this study may be attributed to this phenomenon. Because of relatively high pH (8.0–9.9) and Ca^2+^ concentrations (19–65 mg/ L), and unique microbial compositions in limestone caves [44,65,66], the degradation of eDNA may be accelerated in this study, potentially lowering eDNA detection rates [67].

Second, the timing of major eDNA detection aligned well with adult emergence. In our results, when eDNA detection was evident, males and ovulated and oviposited females were present, such as between May 14 and June 12, and between November 13 and January 10, 2024. In contrast, eDNA detection between January 21 and March 6, 2025, was positive, although adult emergence was not confirmed in the population survey. The erratically large peak at the second site on February 20, as well as those on January 21 and February 7, may have been caused by dead larvae noted on January 21, 2025. The water geochemical changes such as increased biological oxygen demand (BOD), total organic carbon (TOC), and total nitrogen (TN) following extreme winter droughts were responsible for the mortality in this case [68]. As they might gradually decompose, considerable amounts of DNA may be introduced into the primary stream system, resulting in erratic eDNA detection [68]. eDNA detection in late January and February 2005 may be because of post-breeding adults remaining at the breeding site and occasionally entering the water. After winter breeding in December, they cannot move back to their terrestrial habitats in January because winter temperatures significantly drop below freezing in the Korean montane valleys. In any of the *Onychodactylus* species, activities of adults during the non-breeding season have not been studied. Despite these variations, the eDNA detection pattern well reflects that of the emergence pattern of the primary adult. Third, the major eDNA detection patterns were similar between the two eDNA sampling sites; however, the eDNA detection rate at the outlet site was approximately twice as high. In addition, the amount of eDNA detected was approximately 10-fold higher (refer to the scale in Fig 2). These results can be attributed to two factors: 1) many adults may have been present between the first and second eDNA sampling sites. Breeding and post-breeding adults may remain below the first spawning site in areas downstream of the primary spawning site. To approach breeding sites, *Onychodactylus* adults often travel upstream instead of downstream [45]. 2) The first eDNA sampling site was slightly remote from the primary water flow and likely contained less eDNA. The second sampling site, located at the cave outlet, may have gathered large amounts of eDNA from inside the cave. In mountain valleys, eDNA detection is more reliable in low-gathering areas [18,49,69]. Our results indicate that eDNA sampling where water gathers are more efficient, and eDNA detection at more than two locations is advantageous for the appropriate screening of eDNA fluctuations in mountain streams. Overall, a major eDNA detection pattern reflects adult emergence at breeding sites throughout the year but does not accurately reflect larval influx. Abrupt disturbances and post-breeding individuals remaining at the breeding site can lead to unexpected eDNA detection. Any disturbances or events were minimized and carefully recorded during the study. Such information is useful to explain erratic eDNA detection as a post hoc explanation, as shown in this study.

*Onychodactylus koreanus* might breed twice annually. This can be suggested by our three findings. Monitoring the influx of 1-year-old larvae allowed us to roughly track the breeding period. In the present study, eggs hatched within 130 d, which is similar to the known 3–5 months [21,28]. Considering the two major influxes of 1-year-old larvae, the breeding season can be twice annually, and the periods may be estimated to be between May and September, and November and February. The frequency of breeding seasons can be correct; however, the estimated breeding period was too wide, indicating that we could not use larval surveys to determine the period of the breeding season. The time to hatching and yolk absorption may vary depending on the focal spawning site as mentioned above. Therefore, surveying the 1-year-old larval influx cannot adequately track the breeding period. Second, the emergence pattern of ovulated and oviposited females at the breeding sites suggested that *O. koreanus* breeds twice annually, with breeding seasons in May–June and November–December. The spawning of *O. koreanus* in June was previously confirmed in this cave [19], and ovulated *O. koreanus* females moved to the breeding valley in late October in the Hawak mountain [70]. In this study, *O. koreanus* adults were not observed between July and September or between late January and early March, suggesting that the two breeding periods might be separate. Third, the eDNA results also suggest that *O. koreanus* breeds twice annually, in April–June and November–December. During intense mating competition [19], *O. koreanus* may release large amounts of sperm or bodily fluids, similar to fish [10,71]. Therefore, the period of evident eDNA detection in this study might be aligned with the actual breeding events and the occurrence of spawning at the breeding sites of *O. koreanus*. In amphibians, eDNA analysis has been used to detect breeding season, despite variations [13–15]. Researchers have determined the spawning events of fishes by analyzing the ratio of nuclear to mitochondrial DNA detection in eDNA samples [72–74], so we may apply this approach to verify the spawning events of this externally fertilizing subterranean species. Fourth, the discovery of eggs laid on December 23 shows that *O. koreanus* bred during early winter. In 2005 and 2025, more than 100 eggs were observed at the primary spawning site in late spring or early summer [19]. The number of eggs found at the main spawning site was greatly different between 2024 and 2025. Although the clutch size of amphibians in temporal regions largely fluctuated year to year [75], other possibilities, such as the annual shifts of spawning sites, should be questioned. In addition, we confirmed that the larvae and adults of various sizes, which might have been born during different breeding seasons, were all the same species of *O*. *koreanus* based on the COI sequences. Integrated analysis of adult emergence, particularly of ovulated and oviposited females, and the corresponding eDNA analysis allowed reliable estimation of the breeding frequency and period of *O. koreanus*, a subterranean breeder. Our results suggested that *O. koreanus* breeds twice annually, with breeding periods between April and June and November and December.

There are two primary questions regarding the existence of two breeding seasons annually: can a single female breed twice annually, and how do the two breeding seasons occur annually in a population? The annual breeding frequency of an individual largely depends on its energy acquisition and nutritional status [4,76]. Moreover, egg and clutch sizes are also important [77]. In general, larger eggs require more time to undergo vitellogenesis. *Desmognathus folkertsi*, with an egg size of over 4 mm, requires 14–16 months to develop [76]. Thus, female *D. monticola* of the same genus often breeds once every two years. The egg size of *Onychodactylus spp*. is similar to that of plethodontid salamanders: 3.2–5.9 mm in *O. japonicus* [25], 4.9–7.2 mm in *O. koreanus* [19], and 6.7–8.9 mm in *O. fischeri* [27]. The size of these eggs indicates that the development of *Onychodactylus* eggs may require more than 1 year. The habitats of *Onychodactylus spp*. are limited to streams in montane valleys where food sources are limited [78–79]. Therefore, females that lay eggs may require more time to recover their nutritional status. Considering these conditions, it is highly unlikely that a single female lays eggs twice annually. Considering these results, the same female *O. koreanus* is unlikely to participate in either late spring or early winter breeding. The question remains regarding how these two breeding seasons occur annually within a population. This may be because of the combined effects of the two proximate factors, temperature and nutritional conditions. In temperate climatic zones, late May and late October often exhibit similar photoperiods and temperatures. Some amphibians, such as *Pelophylax nigromaculatus* and *P. sphenocephala,* reached their reproductive state during both periods [80–81]. In terms of breeding temperature, *Onychodactylus spp.* may also have two breeding windows in late spring and early winter because early summer and late autumn showed similar breeding temperatures of approximately 3–7 °C [45]. As mentioned above, *Onychodactylus* females may not be able to develop large eggs annually [41]. Therefore, females might use one of the two temperature windows for breeding when they reach reproductive conditions. This hypothesis can be verified by tagging females and monitoring their participation in spawning over time based on their nutritional status or health. However, individuals may regularly use specific breeding periods because of genetic adaptations. It would be interesting to investigate the genetic differentiation and isolation between spring and winter breeding individuals using molecular markers such as nuclear DNA and single-nucleotide polymorphic (SNP) primers.

In conclusion, our results suggest that *O. koreanus* breeds twice annually, from April to June and November to December, and showed that the combined analysis of adult emergence and major eDNA detection patterns could compensate for each other and is useful to predict the breeding frequency and period of a subterranean amphibian breeder. Our approach can also be applied to other studies on subterranean amphibians. Considering that breeding occurs twice annually, various ecological and evolutionary studies on this phenomenon are possible. A broad understanding of the precise breeding season would be helpful for conserving *Onychodactylus spp*. in headwater streams by determining conservation management practices.

## Supporting information

S1 Fig Results of applying different annealing temperatures during PCR runs (57 °C, 60 °C, and 63°C) to determine the optimal annealing temperature in qPCRs using the genomic DNA of two *Onychodactylus koreanus* individuals. The three conditions showed similar amplification results.
(DOCX)

S2 Fig Amplification plots produced in the in vitro tests of the developing primer and probe set using tissue DNAs of the nine amphibian and three reptile species (S2 Table). Only those of *Onychodactylus koreanus* and *O. sillanus* showed significant amplification, but with evidently different CT values.
(DOCX)

S3 Fig Amplification plot produced using different concentrations of the gBlock (10^8–10^-1 copy/μL; S4 Table) during calculating the LOD and LOQ of the developing primer and probe to detect *Onychodactylus koreanus* in environmental DNA (eDNA) samples. (DOCX)

S4 Fig Standard curve of the developing primer and probe set to detect *Onychodactylus koreanus* in environmental DNA (eDNA) samples, produced using different concentrations of the gBlock (10^8–10^-1 copy/μL; S4 Table, S3 Fig) following Klymus et al. (2020). The LOD and LOQ were both 4.52 copies/μL.
(DOCX)

S5 Fig The number of adults (A, B) and larvae (E, F) of *Onychodactylus koreanus* recorded and the amounts of eDNA detection (C, D) at two survey areas and two eDNA sampling sites (A, C, and E vs. B, D, and F) over 14 months between April 2024 and June 2025. (DOCX)

S1 Table List of the species and GenBank accession numbers used in the in-silico tests of the developing primer and probe set for detecting *Onychodactylus koreanus* in environmental DNA (eDNA) samples, with amplification results. (DOCX)

S2 Table The concentration ratio of the probe and primer, developed to detect *Onychodactylus koreanus* in environmental DNA (eDNA) samples, was tested to optimize the relative concentration of the probe to the primer in the qPCR amplification solution, with the CT value results. (DOCX)

S3 Table List of the amphibian and reptile species used in the tissue DNA tests of the developing primer and probe set for detecting *Onychodactylus koreanus* in environmental DNA (eDNA) samples, with amplification results. (DOCX)

S4 Table The sequence information of the gBlock used in this study (176 bp). (DOCX)

S5 Table The concentrations of the gBlock, which are used to determine the LOD and LOQ of the developing primer and probe set for detecting *Onychodactylus koreanus* in environmental DNA (eDNA) samples. (DOCX)

S6 Table The species identification results of the COI sequences, which were amplified from three test and ten experimental eDNA samples using the developed primer and probe set. The amplified sequences were all identified as *Onychodactylus koreanus*.
(DOCX)

S7 Table The number of adults and larvae of *Onychodactylus koreanus* recorded at each of the two survey areas over 14 months (1 and 2) between April 2024 and June 2025. (DOCX)

S8 Table Results (frequency and amounts) of the eDNA detection of *Onychodactylus koreanus* at the two eDNA sampling sites over 14 months (1 and 2) between April 2024 and June 2025. (DOCX)
