# Supplementary material for: Integrated environmental DNA analysis and population assessment revealed a biannual breeding season of the Korean clawed salamander (Onychodactylus koreanus)
Source: PLoS One. 2026 Feb 5;21(2):e0342469. doi: 10.1371/journal.pone.0342469 (PMC12875514; doi:10.1371/journal.pone.0342469)

**Supporting Information**

**S1 Fig. Results of applying different annealing temperatures during PCR runs (57 ℃, 60 ℃, and 63℃) to determine the optimal annealing temperature in qPCRs using the genomic DNA of two *Onychodactylus koreanus* individuals (OK1 and OK2). The three conditions showed similar amplification results.**


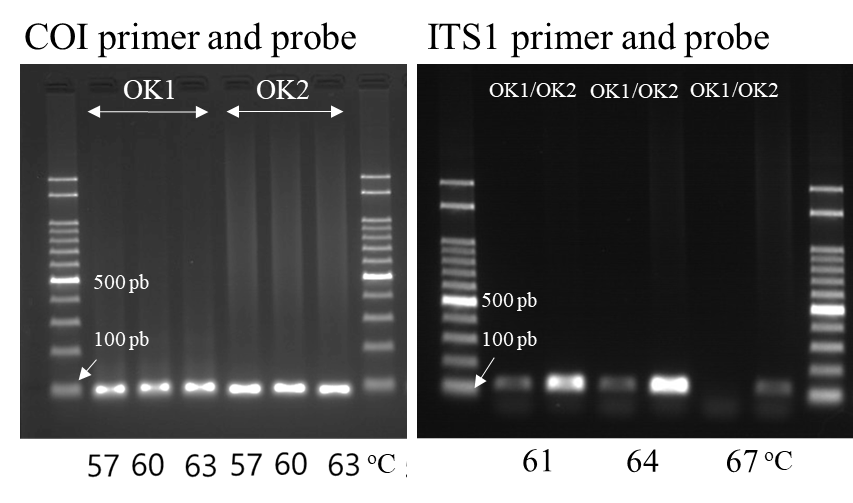

Supplement: S1 Fig — The three conditions showed similar amplification results. (DOCX) [file pone.0342469.s001.docx]
