# Supplementary material for: Integrated environmental DNA analysis and population assessment revealed a biannual breeding season of the Korean clawed salamander (Onychodactylus koreanus)
Source: PLoS One. 2026 Feb 5;21(2):e0342469. doi: 10.1371/journal.pone.0342469 (PMC12875514; doi:10.1371/journal.pone.0342469)

**Supporting Information**

**S2 Fig. Amplification plots produced in the in vitro tests of the developing primer and probe set using tissue DNAs of the nine amphibian and three reptile species (S2 Table). Only those of *Onychodactylus koreanus* and *O. sillanus* showed significant amplification, but with evidently different CT values.**


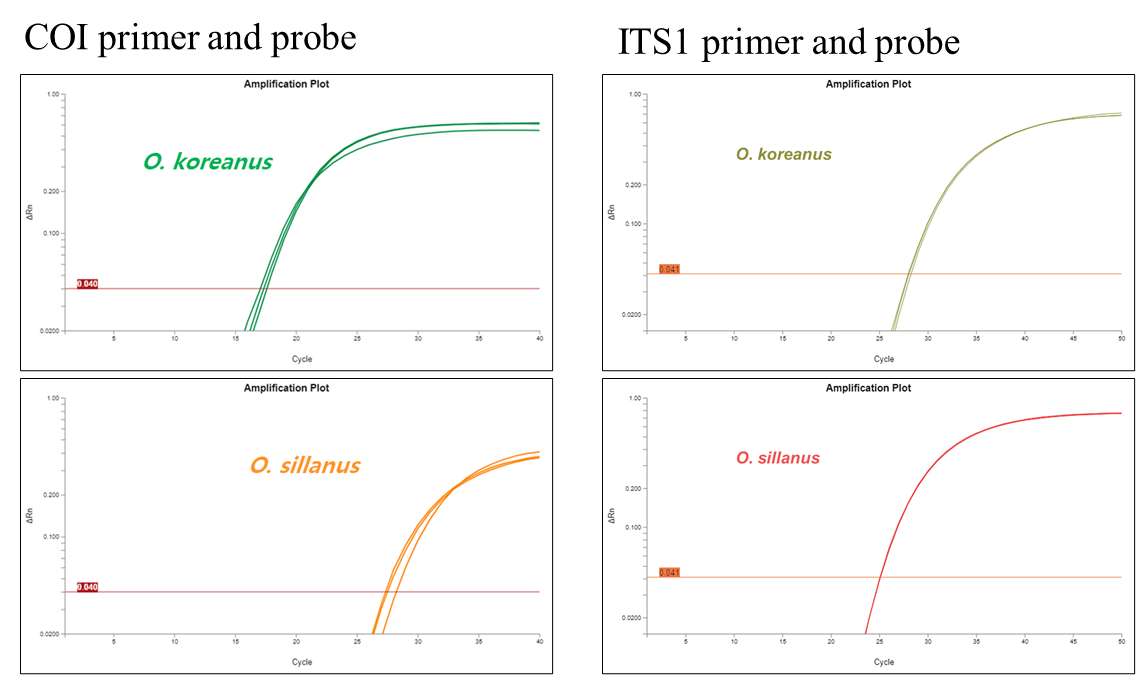

Supplement: S2 Fig — Only those of Onychodactylus koreanus and O. sillanus showed significant amplification, but with evidently different CT values. (DOCX) [file pone.0342469.s002.docx]
