# Supplementary material for: Integrated environmental DNA analysis and population assessment revealed a biannual breeding season of the Korean clawed salamander (Onychodactylus koreanus)
Source: PLoS One. 2026 Feb 5;21(2):e0342469. doi: 10.1371/journal.pone.0342469 (PMC12875514; doi:10.1371/journal.pone.0342469)

**Supporting Information**

**S3 Fig. Amplification plot produced using different concentrations of the gBlock (10^8 –10^-1 copy/μL; S4 Table) during calculating the LOD and LOQ of the developing primer and probe to detect *Onychodactylus koreanus* in environmental DNA (eDNA) samples.**


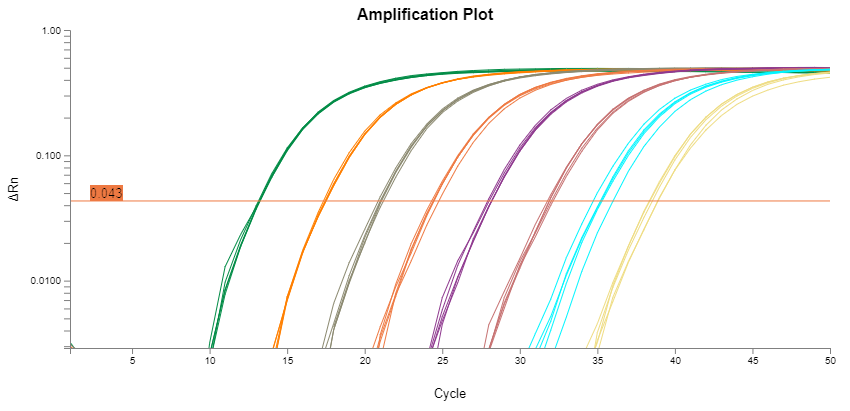

Supplement: S3 Fig — (DOCX) [file pone.0342469.s003.docx]
