# Supplementary material for: Integrated environmental DNA analysis and population assessment revealed a biannual breeding season of the Korean clawed salamander (Onychodactylus koreanus)
Source: PLoS One. 2026 Feb 5;21(2):e0342469. doi: 10.1371/journal.pone.0342469 (PMC12875514; doi:10.1371/journal.pone.0342469)

**Supporting Information**

**S4 Fig. Standard curve of the developing primer and probe set to detect *Onychodactylus koreanus* in environmental DNA (eDNA) samples, produced using different concentrations of the gBlock (10^8–10^-1 copy/μL; S4 Table, S3 Fig) following Klymus et al. (2020). The LOD and LOQ were both 4.52 copies/μL.**


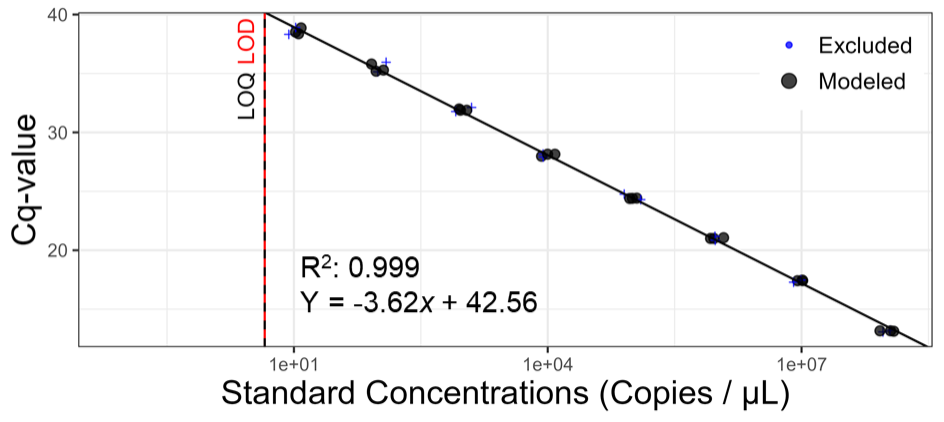

Supplement: S4 Fig — The LOD and LOQ were both 4.52 copies/μL. (DOCX) [file pone.0342469.s004.docx]
