# Supplementary material for: Integrated environmental DNA analysis and population assessment revealed a biannual breeding season of the Korean clawed salamander (Onychodactylus koreanus)
Source: PLoS One. 2026 Feb 5;21(2):e0342469. doi: 10.1371/journal.pone.0342469 (PMC12875514; doi:10.1371/journal.pone.0342469)

**Supporting Information**

**S5 Fig. The number of adults (A, B) and larvae (E, F) of *Onychodactylus koreanus* recorded and the amounts of eDNA detection (C, D) at two survey areas and two eDNA sampling sites (A, C, and E vs. B, D, and F) over 14 months between April 2024 and June 2025.**


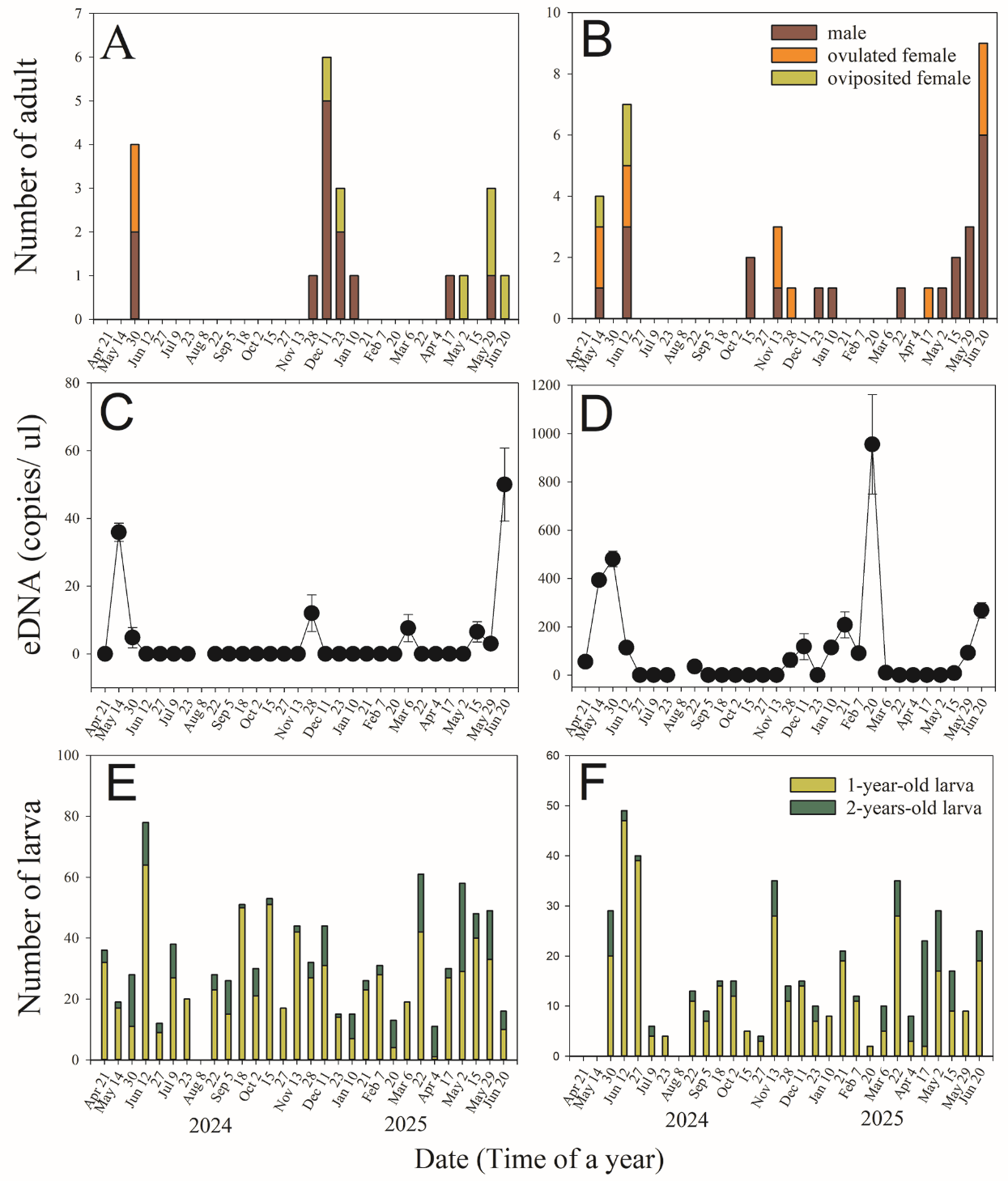

Supplement: S5 Fig — (DOCX) [file pone.0342469.s005.docx]
