# Supplementary material for: Integrated environmental DNA analysis and population assessment revealed a biannual breeding season of the Korean clawed salamander (Onychodactylus koreanus)
Source: PLoS One. 2026 Feb 5;21(2):e0342469. doi: 10.1371/journal.pone.0342469 (PMC12875514; doi:10.1371/journal.pone.0342469)
Supplement: S1 Table — (DOCX) [file pone.0342469.s006.docx]

**Supporting Information**

**S1 Table. List of the species and GenBank accession numbers used in the in-silico tests of the developing primer and probe set for detecting *Onychodactylus koreanus* in environmental DNA (eDNA) samples, with amplification results.**

| Taxon | Species | GenBank accession number | Results |
| --- | --- | --- | --- |
| Amphibian  (11 species; 154 sequences) | *Bombina orientalis* | NC006689 | FAILED |
|  | *Bufo gargarizans* | DQ275350, KM587710, KU321581, C008410 | FAILED |
|  | *Bufo stejnegeri* | KR136211, NC027686 | FAILED |
|  | *Dryophytes japonicus* | NC010232, AB303949 | FAILED |
|  | *Hynobius leechii* | DQ333811, NC008079 | FAILED |
|  | *Karsenia koreana* | JQ844497, JQ844498, JQ844499, MK512368, MT106801, MT106802, MT106803, MT106804, MT106805, MT106806, MT106807, MT106808, MT106809, MT106810, MT106811, MT106812, MT106813, MT106814, MT106815, MT106816, MT106817, MT106818, MT106819, MT106820, MT106821, MT106822, MT106823, MT106824, MT106825 | FAILED |
|  | *Pelophylax nigromaculatus* | AB043889, KT878718 | FAILED |
|  | *Rana coreana* | MF149927, MF149928, MT418666, NC068259 | FAILED |
|  | *Rana huanrensis* | JF939072, JQ844523, JQ844524, JQ844525, JQ844526, KX024951, KX897585, KX897586, KX897587, KX897588, KX897589, KX897590, KX897591, KX897592, KX897593, KX897594, KY030879, KY030880, KY030881, KY030882  KY030883, KY030884, KY030885, KY030886, KY030887, KY030888, KY030889, KY196248, KY196249, KY196250, KY196251, KY196252, KY196253, KY196254, KY196255, KY196256, KY196257, KY196258, KY196259, KY196260  KY196261, KY196262, KY196263, KY196264, KY196265, KY196266, KY196267, KY196268, KY196269, KY196270, KY196271, KY196272, KY196273, KY196274, KY196275, KY196276, KY196277, KY196278, KY196279, KY196280  KY385856, KY385857, KY385858, KY385859, KY385860, KY385861, KY385862, KY385863, KY385864, KY385865, KY385866 KY385867, KY385868, KY385869, MZ779046, NC028521 | FAILED |
|  | *Glandirana rugosa* | AB511301 | FAILED |
|  | *Rana uenoi* | KX024943, KX024944, KX024945, NC056272 | FAILED |
|  | *Elaphe dione* | NC041068 | FAILED |
| Reptile  (7 species; 14 sequences) | *Elaphe schrenckii* | NC027605, NC027605 | FAILED |
|  | *Gloydius brevicaudus* | NC011390 |  |
|  | *Gloydius intermedius* | NC025666, KM434236, MW143075, NC025666 |  |
|  | *Gloydius ussuriensis* | KP262412, NC026553 |  |
|  | *Lycodon rufozonatus* | KF148622, KJ179950 |  |
|  | *Rhabdophis lateralis* | NC030210 |  |
