# Supplementary material for: Integrated environmental DNA analysis and population assessment revealed a biannual breeding season of the Korean clawed salamander (Onychodactylus koreanus)
Source: PLoS One. 2026 Feb 5;21(2):e0342469. doi: 10.1371/journal.pone.0342469 (PMC12875514; doi:10.1371/journal.pone.0342469)
Supplement: S2 Table — (DOCX) [file pone.0342469.s007.docx]

**Supporting Information**

**S2 Table. The concentration ratio of the probe and primer, developed to detect *Onychodactylus koreanus* in environmental DNA (eDNA) samples, was tested to optimize the relative concentration of the probe to the primer in the qPCR amplification solution, with the CT value results.**

| Probe concentration  (nM) | Primer concentration  (nM) | CT value | |
| --- | --- | --- | --- |
|  |  | Mean | Standard Error |
| 250 | 500 | 22.42 | 0.48 |
| 200 | 500 | 21.69 | 0.20 |
| 150 | 500 | 21.93 | 0.06 |
