# Supplementary material for: Integrated environmental DNA analysis and population assessment revealed a biannual breeding season of the Korean clawed salamander (Onychodactylus koreanus)
Source: PLoS One. 2026 Feb 5;21(2):e0342469. doi: 10.1371/journal.pone.0342469 (PMC12875514; doi:10.1371/journal.pone.0342469)
Supplement: S3 Table — (DOCX) [file pone.0342469.s008.docx]

**Supporting Information**

**S3 Table. List of the amphibian and reptile species used in the tissue DNA tests of the developing primer and probe set for detecting *Onychodactylus koreanus* in environmental DNA (eDNA) samples, with amplification results.**

| Taxon | Species | Results |
| --- | --- | --- |
| Amphibian  (9 species) | *Onychodactylus koreanus* | DETECT |
|  | *Onychodactylus sillanus* | DETECT |
|  | *Bombina orientalis* | FAILED |
|  | *Bufo gargarizans* | FAILED |
|  | *Dryophytes japonicus* | FAILED |
|  | *Hynobius leechii* | FAILED |
|  | *Pelophylax nigromaculatus* | FAILED |
|  | *Rana huanrenensis* | FAILED |
|  | *Rana uenoi* | FAILED |
| Reptile  (3 species) | *Elaphe dione* | FAILED |
|  | *Gloydius ussuriensis* | FAILED |
|  | *Rhabdophis lateralis* | FAILED |
