# Supplementary material for: Integrated environmental DNA analysis and population assessment revealed a biannual breeding season of the Korean clawed salamander (Onychodactylus koreanus)
Source: PLoS One. 2026 Feb 5;21(2):e0342469. doi: 10.1371/journal.pone.0342469 (PMC12875514; doi:10.1371/journal.pone.0342469)
Supplement: S4 Table — (DOCX) [file pone.0342469.s009.docx]

**Supporting Information**

**S4 Table. The sequence information of the gBlock used in this study (176 bp).**

| gBlock | Sequence length | Sequence( |
| --- | --- | --- |
|  | 176bp | 5’-GTTTTTGATTATTACCCCCATCATTTCTTCTTTTATTAGCCTCAT  CAGGAGTTGAAGCAGGAGCCGGTACAGGATGAACTGTTTATCCTCCTCTAGCAGGAAATTTAGCACATGCCGGAGCTTCTGTAGACTTAACTATTTTTTCTTTACACTTAGCAGGCATTTCATCAATTCTA-3’ |
