# Supplementary material for: Integrated environmental DNA analysis and population assessment revealed a biannual breeding season of the Korean clawed salamander (Onychodactylus koreanus)
Source: PLoS One. 2026 Feb 5;21(2):e0342469. doi: 10.1371/journal.pone.0342469 (PMC12875514; doi:10.1371/journal.pone.0342469)
Supplement: S5 Table — (DOCX) [file pone.0342469.s010.docx]

**Supporting Information**

**S5 Table. The concentrations of the gBlock, which are used to determine the LOD and LOQ of the developing primer and probe set for detecting *Onychodactylus koreanus* in environmental DNA (eDNA) samples.**

| gBlock sample  (copy/ μL) | CT  (Five replicates) | Quantity |
| --- | --- | --- |
| 10^8 | 13.13162, 13.07409, 13.20741  13.16568, 13.16622 | 100000000 |
| 10^7 | 17.46585, 17.42437, 17.47293  17.41906, 17.29109 | 10000000 |
| 10^6 | 21.06468, 21.00306, 21.18246  21.00522, 20.85461 | 1000000 |
| 10^5 | 24.76279, 24.42317, 24.40692  24.39493, 24.29483 | 100000 |
| 10^4 | 27.981, 28.15709, 27.9105  28.14889, 28.15043 | 10000 |
| 10^3 | 31.98595, 32.1174, 31.88125  31.77177, 31.88256 | 1000 |
| 10^2 | 35.13953, 35.94961, 35.19311  35.28545, 35.79994 | 100 |
| 10^1 | 38.31736, 38.86486, 38.37126  38.50998, 38.9013 | 10 |
| 10^0 | No detection | 1 |
| 10^-1 | No detection | 0.1 |
