# Supplementary material for: Integrated environmental DNA analysis and population assessment revealed a biannual breeding season of the Korean clawed salamander (Onychodactylus koreanus)
Source: PLoS One. 2026 Feb 5;21(2):e0342469. doi: 10.1371/journal.pone.0342469 (PMC12875514; doi:10.1371/journal.pone.0342469)
Supplement: S6 Table — The amplified sequences were all identified as Onychodactylus koreanus. (DOCX) [file pone.0342469.s011.docx]

**Supporting Information**

**S6 Table. The species identification results of the COI sequences, which were amplified from three test and ten experimental eDNA samples using the developed primer and probe set. The amplified sequences were all identified as *Onychodactylus koreanus*.**

| Sample  type | Locality | Cq value | Sampling  Date  (YYMMDD) | Sequence | Per. of identification in the NCBI BLAST | Identified species |
| --- | --- | --- | --- | --- | --- | --- |
| Test  sample | Goeun, Chuncheon | 37.38 | 240327 | 5’‑AGCCTCATCAGGAGTTGAAGCAGGAGCCGGTACAGGATGAACNNNNNNNCCTCCTCTAGCAGGAAATTTAGCACATGCCGGAGCTTCTGTAGA-3’ | 92.47 | *O. koreanus* |
|  | Doekduwon, Chuncheon | 34.51 | 240329 | 5’‑AGCCTCATCAGGAGTTGAAGCAGGAGCCGGTACAGGATGAACNGTTTATCCTCCTCTAGCAGGAAATTTAGCACATGCCGGAGCTTCTGTAGA-3’ | 98.92 | *O. koreanus* |
|  | Neuratjae, Chuncheon | 35.47 | 240509 | 5’‑AGCCTCATCAGGAGTTGAAGCAGGAGCCGGTACAGGATGAACTGTTTATCCTCCTCTAGCAGGAAATTTAGCACATGCCGGAGCTTCTGTAGA-3’ | 100 | *O. koreanus* |
| Exp.  sample | Study site 1 | 38.70 | 240514 | 5’‑AGCCTCATCAGGAGTTGAAGCAGGAGCCGGTACAGGATGAACTGTTTATCCTCCTCTAGCAGGAAATTTAGCACATGCCGGAGCTTCTGTAGA-3’ | 100 | *O. koreanus* |
|  |  | 38.88 | 240530 | 5’‑AGCCTCATCAGGAGTTGAAGCAGGAGCCGGTACAGGATGAACTGTTTATCCTCCTCTAGCAGGAAATTTAGCACATGCCGGAGCTTCTGTAGA-3’ | 100 | *O. koreanus* |
|  |  | 39.42 | 241128 | 5’‑AGCCTCATCAGGAGTTGAAGCAGGAGCCGGTACAGGATGAACTGTTTATCCTCCTCTAGCAGGAANTTTAGCACATGCCGGAGCTTCTGTAGA-3’ | 98.92 | *O. koreanus* |
|  |  | 38.63 | 250515 | 5’‑AGCCTCATCAGGAGTTGAAGCAGGAGCCGGTACAGGATGAACTGTTTATCCTCCTCTAGCAGGAAATTTAGCACATGCCGGAGCTTCTGTAGA-3’ | 100 | *O. koreanus* |
|  |  | 39.58 | 250529 | 5’‑AGCCTCATCAGGAGTTGAAGCAGGAGCCGGTACAGGATGAACTGTTTATCCTCCTCTAGCAGGAAATTTAGCACATGCCGGAGCTTCTGTAGA-3’ | 100 | *O. koreanus* |
|  | Study site 2 | 35.42 | 240514 | 5’‑AGCCTCATCAGGAGTTGAAGCAGGAGCCGGTACAGGATGAACTGTTTATCCTCCTCTAGCAGGAAATTTAGCACATGCCGGAGCTTCTGTAGA-3’ | 100 | *O. koreanus* |
|  |  | 36.56 | 240514 | 5’‑AGCCTCATCAGGAGTTGAAGCAGGAGCCGGTACAGGATGAACTGTTTATCCTCCTCTAGCAGGAAATTTAGCACATGCCGGAGCTTCTGTAGA-3’ | 100 | *O. koreanus* |
|  |  | 35.00 | 240530 | 5’‑AGCCTCATCAGGAGTTGAAGCAGGAGCCGGTACAGGATGAACNNNNNNNCCTCCTCTAGCAGGAAATTTAGCACATGCCGGAGCTTCTGTAGA-3’ | 92.47 | *O. koreanus* |
|  |  | 37.41 | 240530 | 5’‑AGCCTCATCAGGAGTTGAAGCAGGAGCCGGTACAGGATGAACTGTTTATCCTCCTCTAGCAGGAAATTTAGCACATGCCGGAGCTTCTGTAGA-3’ | 100 | *O. koreanus* |
|  |  | 38.09 | 240612 | 5’‑AGCCTCATCAGGAGTNGAAGCAGGAGCCGGTACAGGATGAACTGTTTATCCTCCTCTAGCAGGAANTTTAGCACATGCCGGAGCTTCTGTAGA-3’ | 97.85 | *O. koreanus* |
|  |  | 34.74 | 240822 | 5’‑AGCCTCATCAGGAGTNGAAGCAGGAGCCGGTACAGGATGAACTGNNNNTCCTCCTCTAGCAGGAATTTAGCACATGCCGGAGCTTCTGTAGA -3’ | 93.55 | *O. koreanus* |
|  |  | 38.71 | 241128 | 5’‑AGCCTCATCAGGAGTTGAAGCAGGAGCCGGTACAGGATGAACNGTTTATCCTCCTCTAGCAGGAAATTTAGCACATGCCGGAGCTTCTGTAGA-3’ | 98.92 | *O. koreanus* |
|  |  | 38.39 | 241211 | 5’‑AGCCTCATCAGGAGTTGAAGCAGGAGCCGGTACAGGATGAACTGNNNNTCCTCCTCTAGCAGGAANTTTAGCACATGCCGGAGCTTCTGTAGA-3’ | 94.62 | *O. koreanus* |
|  |  | 36.77 | 250110 | 5’‑AGCCTCATCAGGAGTTGAAGCAGGAGCCGGTACAGGATGAACNNNTTATCCTCCTCTAGCAGGAANTTTAGCACATGCCGGAGCTTCTGTAGA-3’ | 95.70 | *O. koreanus* |
|  |  | 37.06 | 250121 | 5’‑AGCCTCATCAGGAGTTGAAGCAGGAGCCGGTACAGGATGAACTGTTNNTCCTCCTCTAGCAGGAANTTTAGCACATGCCGGAGCTTCTGTAGA-3’ | 96.77 | *O. koreanus* |
|  |  | 36.50 | 250121 | 5’‑AGCCTCATCAGGAGTTGAAGCAGGAGCCGGTACAGGATGAACTGTNNNTCCTCCTCTAGCAGGAANTTTAGCACATGCCGGAGCTTCTGTAGA-3’ | 95.70 | *O. koreanus* |
|  |  | 33.86 | 250220 | 5’‑AGCCTCATCAGGAGTTGAAGCAGGAGCCGGTACAGGATGAACNNNTTATCCTCCTCTAGCAGGAANTTTAGCACATGCCGGAGCTTCTGTAGA-3’ | 95.70 | *O. koreanus* |
|  |  | 39.71 | 250515 | 5’‑AGCCTCATCAGGAGTTGAAGCAGGAGCCGGTACAGGATGAACTGTTTATCCTCCTCTAGCAGGAAATTTAGCACATGCCGGAGCTTCTGTAGA-3’ | 100 | *O. koreanus* |
|  |  | 36.12 | 250529 | 5’‑AGCCTCATCAGGAGTTGAAGCAGGAGCCGGTACAGGATGAACTGTTTATCCTCCTCTAGCAGGAANTTTAGCACATGCCGGAGCTTCTGTAGA-3’ | 98.92 | *O. koreanus* |
|  |  | 38.03 | 250529 | 5’‑AGCCTCATCAGGAGTTGAAGCAGGAGCCGGTACAGGATGAACTGTTTATCCTCCTCTAGCAGGAAATTTAGCACATGCCGGAGCTTCTGTAGA-3’ | 100 | *O. koreanus* |
|  |  | 35.98 | 250620 | 5’‑AGCCTCATCAGGAGTTGAAGCAGGAGCCGGTACAGGATGAACTGTTTATCCTCCTCTAGCAGGAAATTTAGCACATGCCGGAGCTTCTGTAGA-3’ | 100 | *O. koreanus* |
|  |  | 36.99 | 250620 | 5’‑AGCCTCATCAGGAGTTGAAGCAGGAGCCGGTACAGGATGAACTGTTTATCCTCCTCTAGCAGGAAATTTAGCACATGCCGGAGCTTCTGTAGA-3’ | 100 | *O. koreanus* |
