# Supplementary material for: Integrated environmental DNA analysis and population assessment revealed a biannual breeding season of the Korean clawed salamander (Onychodactylus koreanus)
Source: PLoS One. 2026 Feb 5;21(2):e0342469. doi: 10.1371/journal.pone.0342469 (PMC12875514; doi:10.1371/journal.pone.0342469)
Supplement: S7 Table — (DOCX) [file pone.0342469.s012.docx]

**Supporting Information**

**S7 Table. The number of adults and larvae of *Onychodactylus koreanus* recorded at each of the two survey areas over 14 months (1 and 2) between April 2024 and June 2025.**

| Replicate  Date | Survey area 1 | | | | | | | Survey area 2 | | | | | | |
| --- | --- | --- | --- | --- | --- | --- | --- | --- | --- | --- | --- | --- | --- | --- |
|  | Adult | | | | Larva | | | Adult | | | | Larva | | |
|  | Total | Male | Ovulated female | Oviposited female | Total | 1-year-old | 2-years-old | Total | Male | Ovulated female | Oviposited female | Total | 1-year-old | 2-years-old |
| 21-Apr | 0 | 0 | 0 | 0 | 36 | 32 | 4 | no survey | | | | | | |
| 14-May | 4 | 1 | 2 | 1 | 19 | 17 | 2 | no survey | | | | | | |
| 30-May | 0 | 0 | 0 | 0 | 28 | 11 | 17 | 4 | 2 | 2 | 0 | 29 | 20 | 9 |
| 12-Jun | 7 | 3 | 2 | 2 | 78 | 64 | 14 | 1 | 1 | 0 | 0 | 49 | 47 | 2 |
| 27-Jun | 0 | 0 | 0 | 0 | 12 | 9 | 3 | 0 | 0 | 0 | 0 | 40 | 39 | 1 |
| 09-Jul | 0 | 0 | 0 | 0 | 38 | 27 | 11 | 0 | 0 | 0 | 0 | 6 | 4 | 2 |
| 23-Jul | 0 | 0 | 0 | 0 | 20 | 20 | 0 | 0 | 0 | 0 | 0 | 4 | 4 | 0 |
| 08-Aug | no survey | | | | | | | no survey | | | | | | |
| 22-Aug | 0 | 0 | 0 | 0 | 28 | 23 | 5 | 0 | 0 | 0 | 0 | 13 | 11 | 2 |
| 05-Sep | 0 | 0 | 0 | 0 | 26 | 15 | 11 | 0 | 0 | 0 | 0 | 9 | 7 | 2 |
| 18-Sep | 0 | 0 | 0 | 0 | 51 | 50 | 1 | 0 | 0 | 0 | 0 | 15 | 14 | 1 |
| 02-Oct | 0 | 0 | 0 | 0 | 30 | 21 | 9 | 0 | 0 | 0 | 0 | 15 | 12 | 3 |
| 15-Oct | 2 | 2 | 0 | 0 | 53 | 51 | 2 | 0 | 0 | 0 | 0 | 5 | 5 | 0 |
| 27-Oct | 0 | 0 | 0 | 0 | 17 | 17 | 0 | 0 | 0 | 0 | 0 | 4 | 3 | 1 |
| 13-Nov | 3 | 1 | 2 | 0 | 44 | 42 | 2 | 0 | 0 | 0 | 0 | 35 | 28 | 7 |
| 28-Nov | 1 | 0 | 1 | 0 | 32 | 27 | 5 | 1 | 1 | 0 | 0 | 14 | 11 | 3 |
| 11-Dec | 0 | 0 | 0 | 0 | 44 | 31 | 13 | 6 | 5 | 0 | 1 | 15 | 14 | 1 |
| 23-Dec | 1 | 1 | 0 | 0 | 15 | 14 | 1 | 3 | 2 | 0 | 1 | 10 | 7 | 3 |
| 10-Jan | 1 | 1 | 0 | 0 | 15 | 7 | 8 | 1 | 1 | 0 | 0 | 8 | 8 | 0 |
| 21-Jan | 0 | 0 | 0 | 0 | 26 | 23 | 3 | 0 | 0 | 0 | 0 | 21 | 19 | 2 |
| 07-Feb | 0 | 0 | 0 | 0 | 31 | 28 | 3 | 0 | 0 | 0 | 0 | 12 | 11 | 1 |
| 20-Feb | 0 | 0 | 0 | 0 | 13 | 4 | 9 | 0 | 0 | 0 | 0 | 2 | 2 | 0 |
| 06-Mar | 0 | 0 | 0 | 0 | 19 | 19 | 0 | 0 | 0 | 0 | 0 | 10 | 5 | 5 |
| 22-Mar | 1 | 1 | 0 | 0 | 61 | 42 | 19 | 0 | 0 | 0 | 0 | 35 | 28 | 7 |
| 04-Apr | 0 | 0 | 0 | 0 | 11 | 1 | 10 | 0 | 0 | 0 | 0 | 8 | 3 | 5 |
| 17-Apr | 1 | 0 | 1 | 0 | 30 | 27 | 3 | 1 | 1 | 0 | 0 | 23 | 2 | 21 |
| 02-May | 1 | 1 | 0 | 0 | 58 | 29 | 29 | 1 | 0 | 0 | 1 | 29 | 17 | 12 |
| 15-May | 2 | 2 | 0 | 0 | 48 | 40 | 8 | 0 | 0 | 0 | 0 | 17 | 9 | 8 |
| 29-May | 3 | 3 | 0 | 0 | 49 | 33 | 16 | 3 | 1 | 0 | 2 | 9 | 9 | 0 |
| 20-Jun | 9 | 6 | 3 | 0 | 16 | 10 | 6 | 1 | 0 | 0 | 1 | 25 | 19 | 6 |
