# Supplementary material for: Integrated environmental DNA analysis and population assessment revealed a biannual breeding season of the Korean clawed salamander (Onychodactylus koreanus)
Source: PLoS One. 2026 Feb 5;21(2):e0342469. doi: 10.1371/journal.pone.0342469 (PMC12875514; doi:10.1371/journal.pone.0342469)
Supplement: S8 Table — (DOCX) [file pone.0342469.s013.docx]

**Supporting Information**

**S8 Table. Results (frequency and amounts) of the eDNA detection of *Onychodactylus koreanus* at the two eDNA sampling sites over 14 months (1 and 2) between April 2024 and June 2025.**

| Replicate  Date | Sampling site 1 | | | | | Sampling site 2 | | | | |
| --- | --- | --- | --- | --- | --- | --- | --- | --- | --- | --- |
|  | Amounts of  extracted eDNA  (ng/ μL) | | eDNA detection  (/3 replicates) | | Amounts of eDNA detection  (copies/ μL) | Amounts of  extracted eDNA  (ng/ μL) | | eDNA detection  (/3 replicates) | | Amounts of eDNA detection  (copies/ μL) |
|  | 1st set | 2nd set | 1st set | 2nd set | mean±SE | 1st set | 2nd set | 1st set | 2nd set | mean±SE |
| 21-Apr | 4.8 | 4.1 | 0 | 0 | 0.0±0.0 | 2.8 | 2.8 | 2 | 2 | 55.4±17.6 |
| 14-May | 3.1 | 3.6 | 3 | 3 | 35.9±2.7 | 2.6 | 3.8 | 3 | 3 | 393.3±25.2 |
| 30-May | 4.4 | 2.1 | 2 | 0 | 4.8±3.0 | 2.6 | 6.9 | 3 | 3 | 480.7±31.8 |
| 12-Jun | 3.8 | 2.7 | 0 | 0 | 0.0±0.0 | 3.0 | 1.4 | 3 | 2 | 113.8±26.4 |
| 27-Jun | 3.5 | 3.2 | 0 | 0 | 0.0±0.0 | 2.6 | 2.3 | 0 | 0 | 0.0±0.0 |
| 09-Jul | 4.6 | 4.2 | 0 | 0 | 0.0±0.0 | 5.0 | 6.4 | 0 | 0 | 0.0±0.0 |
| 23-Jul | 1.9 | 4.0 | 0 | 0 | 0.0±0.0 | 0.6 | 3.8 | 0 | 0 | 0.0±0.0 |
| 08-Aug | No sampling | | | | | No sampling | | | | |
| 22-Aug | 1.6 | 4.5 | 0 | 0 | 0.0±0.0 | 2.4 | 2.9 | 3 | 3 | 35.2±3.5 |
| 05-Sep | 1.1 | 3.0 | 0 | 0 | 0.0±0.0 | 1.0 | 2.8 | 0 | 0 | 0.0±0.0 |
| 18-Sep | 2.2 | 4.7 | 0 | 0 | 0.0±0.0 | 1.7 | 5.3 | 0 | 0 | 0.0±0.0 |
| 02-Oct | 2.9 | 1.2 | 0 | 0 | 0.0±0.0 | 3.1 | 1.9 | 0 | 0 | 0.0±0.0 |
| 15-Oct | 3.4 | 1.9 | 0 | 0 | 0.0±0.0 | 4.4 | 2.1 | 0 | 0 | 0.0±0.0 |
| 27-Oct | 1.6 | 2.6 | 0 | 0 | 0.0±0.0 | 1.0 | 1.4 | 0 | 0 | 0.0±0.0 |
| 13-Nov | 2.9 | 3.5 | 0 | 0 | 0.0±0.0 | 1.1 | 3.5 | 0 | 0 | 0.0±0.0 |
| 28-Nov | 2.3 | 2.8 | 0 | 3 | 12.0±5.4 | 1.2 | 2.2 | 0 | 3 | 62.5±28.7 |
| 11-Dec | 2.9 | 4.2 | 0 | 0 | 0.0±0.0 | 1.1 | 2.9 | 0 | 3 | 117.9±53.7 |
| 23-Dec | 1.9 | 2.5 | 0 | 0 | 0.0±0.0 | 1.9 | 1.2 | 0 | 0 | 0.0±0.0 |
| 10-Jan | 2.5 | 3.5 | 0 | 0 | 0.0±0.0 | 7.7 | 5.1 | 3 | 3 | 114.2±10.8 |
| 21-Jan | 1.9 | 3.1 | 0 | 0 | 0.0±0.0 | 6.6 | 9.7 | 3 | 3 | 208.2±53.3 |
| 07-Feb | 3.0 | 4.2 | 0 | 0 | 0.0±0.0 | 9.8 | 12.6 | 3 | 3 | 90.4±5.5 |
| 20-Feb | 1.9 | 2.7 | 0 | 0 | 0.0±0.0 | 32.5 | 28.8 | 3 | 3 | 955.2±206.0 |
| 06-Mar | 2.0 | 3.2 | 3 | 0 | 7.6±4.0 | 1.4 | 2.8 | 2 | 0 | 9.7±6.2 |
| 22-Mar | 3.6 | 5.0 | 0 | 0 | 0.0±0.0 | 4.0 | 4.8 | 0 | 0 | 0.0±0.0 |
| 04-Apr | 1.4 | 2.7 | 0 | 0 | 0.0±0.0 | 1.8 | 2.7 | 0 | 0 | 0.0±0.0 |
| 17-Apr | 2.3 | 2.5 | 0 | 0 | 0.0±0.0 | 1.8 | 3.8 | 0 | 0 | 0.0±0.0 |
| 02-May | 2.3 | 4.6 | 0 | 0 | 0.0±0.0 | 1.7 | 2.3 | 0 | 0 | 0.0±0.0 |
| 15-May | 1.8 | 2.7 | 0 | 3 | 6.5±3.0 | 1.9 | 2.4 | 0 | 3 | 8.0±4.3 |
| 29-May | 1.9 | 2.7 | 3 | 0 | 3.0±1.6 | 8.3 | 3.5 | 3 | 2 | 91.9±21.3 |
| 20-Jun | 2.3 | 3.8 | 3 | 3 | 50.0±10.8 | 5.5 | 6.1 | 3 | 3 | 267.9±31.9 |
| Mean±SE | 2.6±0.2 | 3.3±0.2 |  |  | 4.1±1.1 | 4.2±1.1 | 4.8±1.0 |  |  | 103.6±18.2 |
